# Case Report: Bone fragment in the third ventricle of a 22 year-old woman

**DOI:** 10.12688/f1000research.6180.2

**Published:** 2015-03-31

**Authors:** Sunil Munakomi, Balaji Srinivas, Iype Cherian

**Affiliations:** 1 International Society for Medical Education, College of Medical Sciences, Bharatpur, Chitwan, Nepal

**Keywords:** bone fragment, brain surgery, compound-depressed fracture

## Abstract

Here we present a very rare case of a woman with a bone fragment in the third ventricle of the brain following compound-depressed skull fractures due to a road traffic accident.

There are only few case reports of bullets and textiloma being removed from the third ventricle. Following operative removal of the fragment, the patient was started on cortisol, mineralocorticoid and thyroid hormone replacement. However, the patient eventually died of the severe traumatic hypothalamic insult.

## Case report

**Figure 1.**
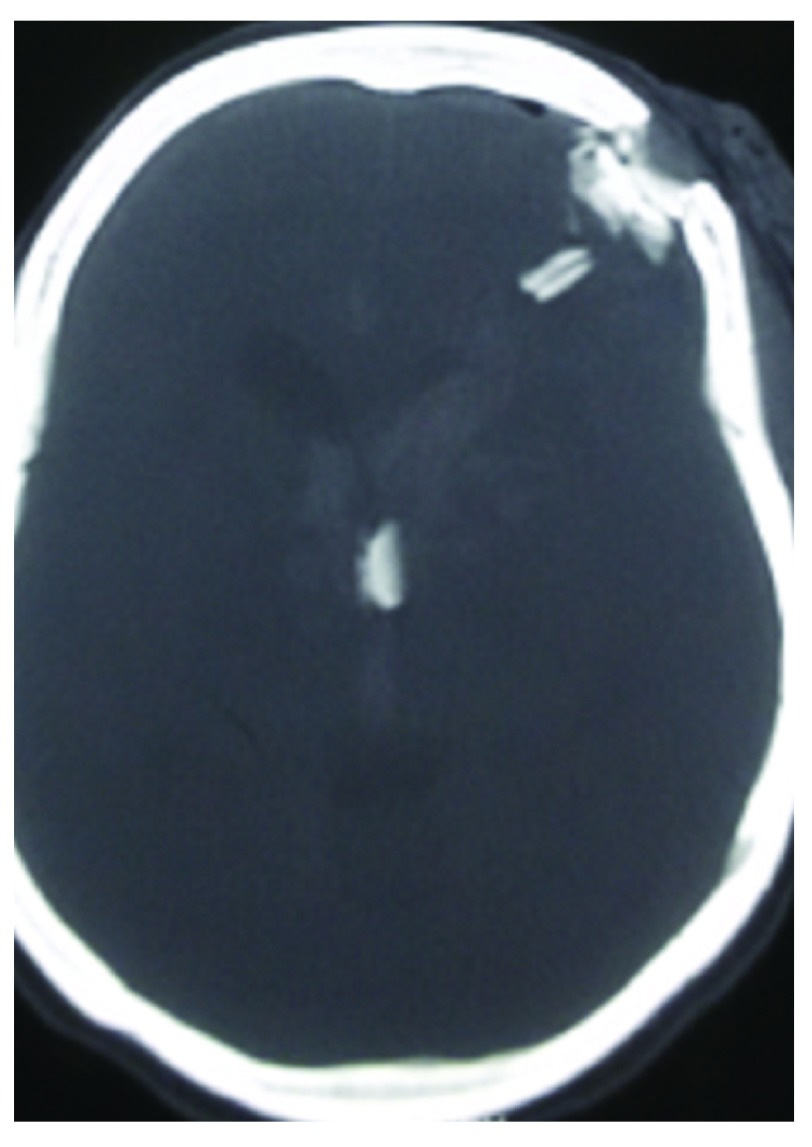
CT image showing the bone fragment lodged in the third ventricle.

**Figure 2.**
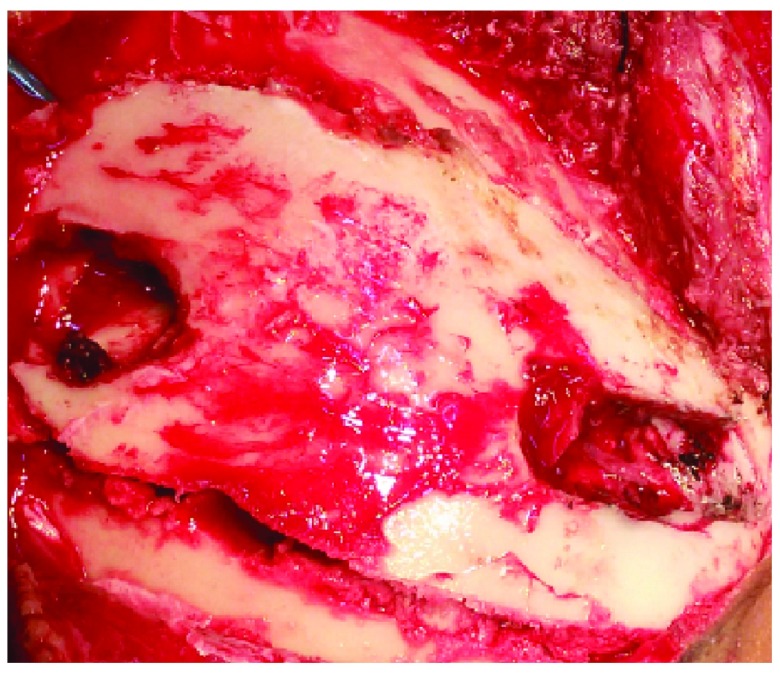
Intra-operative picture showing two sites of compound depressed fracture and the craniotomy performed circling both of them.

**Figure 3.**
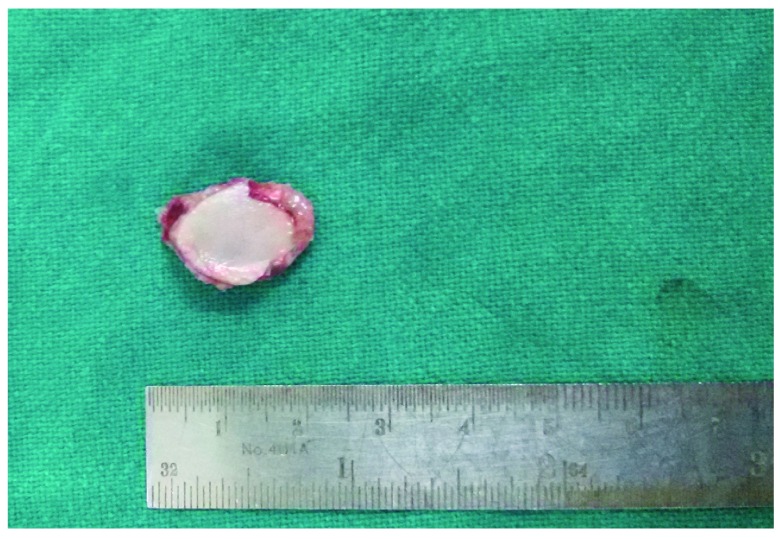
Image showing the bone fragment retrieved from the third ventricle.

**Figure 4.**
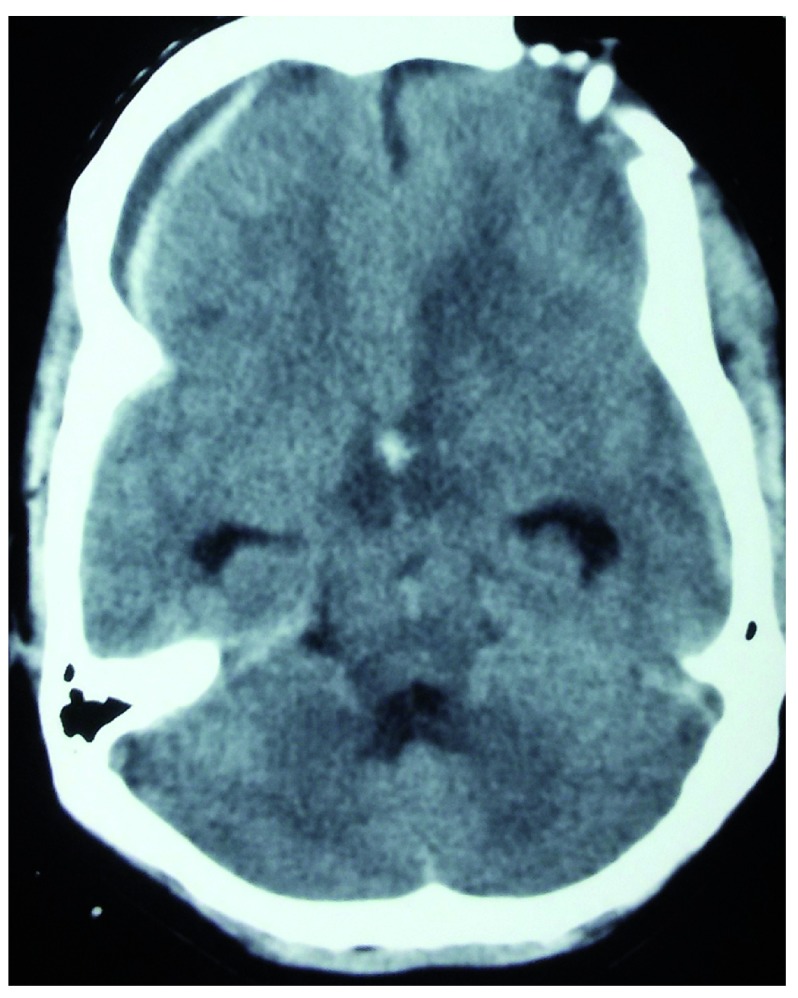
Post-operative image showing evidence of hypodensities surrounding hypothalamic region.

A 22 year-old female, with no significant past medical and surgical illnesses, was brought to the casualty room with a Glasgow coma scale of 6/15 following a collision between two bikes three hours earlier. Local examination revealed two compound depressed skull fractures in the frontal and the parietal region with egress of brain matter. Following primary resuscitation, computed tomography (CT) of the head confirmed the local findings along with the presence of one bone fragment in the third ventricle ( Figure 1). The patient was taken for debridement of the wound and craniotomy circling the depressed sites. Since the patient was already extending and because there was already hemoventriculi, we opted for removal of the fragment despite its anatomical location so as to minimize further damage and chance of hydrocephalus. The bone fragment in the third ventricle was easily accessible following the hematoma track. An endoscope was also kept ready just in case the corridor to the fragment was difficult to access. Following retrieval of the bone fragment ( Figure 2, Figure 3), intraventricular drain was placed and neurosurgical intensive care was provided. Repeated CT scans showed hypodensities around the third ventricle ( Figure 4). On the second post-operative day, the patient was started on ionotropic support because of the refractory hypotension, and was also replaced with hydrocortisone, fludrocortisone and thyroid hormones. Wound dressing and the ventricular drain care was continued. Cerebrospinal fluid (CSF) culture from the drain resulted sterile. The patient died on the 8 ^th^ post-operative day because of the traumatic severe hypothalamic insult.

## Discussion

As brain abscesses may result from driven bone fragments and other retained foreign bodies in the brain, the removal of readily accessible foreign bodies has received much attention ^3– 6^. Migration of foreign bodies can occur because of gravitational force. Other routes of migration can be subdural, parenchymal, transventricular or along streamlining along the white matter track ^7^. The removal of foreign bodies is mostly done via craniotomy ^8, 9^, but other methods such as burr hole, stereotaxy ^10^ and sometimes by ventriculoscopy ^11^ have also been described.

The goals of modern treatments include removal of the foreign body under a controlled environment in the neurosurgical operation setting. Surgical principles include removal of bone fragments, intracerebral hematoma, control of hemorrhages and prevention of further loss of neural tissue. Patients should receive a broad spectrum intravenous antibiotic therapy along with tetanus prophylaxis. Monitoring and control of elevated intracranial pressure with maintenance of cerebral perfusion pressure plays a significant role in the patient’s survival and outcome. The follow-up of such patients is essential, considering known complications like cerebrospinal fluid fistula in the early post-operative period and brain abscesses and seizures which may occur years after injury. Outcome after a penetrating head injury is directly related to the Glasgow coma scale at the time of presentation, which is the reflection of the extent of brain tissue damage caused directly by the primary impact. Intensive post-operative monitoring of intracranial pressure, cardio-respiratory function and metabolic status are required for optimizing the outcome of victims of penetrating craniocerebral injuries ^12^. Penetrating head injuries have a higher mortality and morbidity than blunt trauma even in a civilian set up ^13^. Even after timely removal of the penetrating objects and intensive medical management, the outcome may remain poor.

## Consent

Informed written consent for publication of images and clinical details was obtained from the patient’s husband.
