# Supplementary material for: Case Report: Bone fragment in the third ventricle of a 22 year-old woman
Source: F1000Res. 2015 Mar 31;4:63. Originally published 2015 Mar 11. [Version 2] doi: 10.12688/f1000research.6180.2 (PMC4560254; doi:10.12688/f1000research.6180.2)
Supplement: Peer review history

# Peer review: Referee response for version 2

**DOI:** 10.5256/f1000research.6785.r8885

**Published:** 2015-07-07

**Authors:** Guoyi Gao

**Affiliations:** 1 Department of Neurosurgery, Renji Hospital, Shanghai Institute of Head Trauma, Shanghai, China

I read this report with high interests not only for this is a rare case of bone fragment moved into the third ventricle, I am also impressed by the surgical efforts to remove it. Obviously it is a successful operation and it indicates the well trained neurosurgical technique of the surgeon. The fatal outcome resulted from the failure of hypothalamus function, which is still a problem to be solved in the future.

I have read this submission. I believe that I have an appropriate level of expertise to confirm that it is of an acceptable scientific standard.

# Peer review: Referee response for version 2

**DOI:** 10.5256/f1000research.6785.r8705

**Published:** 2015-05-27

**Authors:** Ajit Shrestha

**Affiliations:** 1 National Institute of Neurological and Allied Sciences, Kathmandu, Nepal

This is a rare case report from civilian society and is obviously will invite mixed school of thoughts.

The authors have wise reasons, obstructive hydrocephalus and infection, for removing the third ventricular bone fragment and, in doing so, has stated that the hematoma track, formed by the fragment traversing through the parenchyma, has been followed. The authors had made prudent decision by keeping neuroendoscope in hand for the removal of fragment in case of complications.

This report, I personally feel, encourages all trauma neurosurgeons to attempt removal of foreign body or possible source of infection or obstruction if it is accessible without undue further trauma to parenchyma.

I have read this submission. I believe that I have an appropriate level of expertise to confirm that it is of an acceptable scientific standard.

# Peer review: Referee response for version 1

**DOI:** 10.5256/f1000research.6625.r7934

**Published:** 2015-03-24

**Authors:** Andrey Belkin, Alexey Pychteev

**Affiliations:** 1 Clinical Institute of the Brain, Ekaterinburg, Sverdlovsk region, Russian Federation

It seems obvious that surgery even on symptomatic intracerebral foreign bodies is determined by the anatomical availability and physiological fact. In this case, initially, it seems that the risk of surgery was higher than the risk of complications due to the presence of a foreign body and, therefore, the intervention was not justified.

In order to give a more detailed review we would need to have a tomogram data on the patient’s condition. Based on the currently available outline of the situation, this patient would require more conservative treatment (aggressive antibiotic therapy in the presence of the slightest signs of SIRS or antibiotic prophylaxis from the first hours).

Only if the patient on admission had a detailed diencephalic syndrome (and judging by the description they did not) and clinical signs of meningitis, would we try endoscopic removal of the foreign body (if it is of small size, somewhere up to 1 cm). Thus one alternative conservative intervention is to attempt endoscopic evacuation.

If endoscopic evacuation is not possible (large size foreign bodies, technical inaccessibility through the ventricle), another possible surgical action that I would have done (but only in the case of ventriculitis) is external ventricular drainage. No more.

We have read this submission. We believe that we have an appropriate level of expertise to state that we do not consider it to be of an acceptable scientific standard, for reasons outlined above.
